# Genome-wide identification of mitogen-activated protein kinase gene family in *Gossypium raimondii* and the function of their corresponding orthologs in tetraploid cultivated cotton

**DOI:** 10.1186/s12870-014-0345-9

**Published:** 2014-12-10

**Authors:** Xueying Zhang, Liman Wang, Xiaoyang Xu, Caiping Cai, Wangzhen Guo

**Affiliations:** State Key Laboratory of Crop Genetics & Germplasm Enhancement, Hybrid Cotton R & D Engineering Research Center, MOE, Nanjing Agricultural University, Nanjing, 210095 Jiangsu Province P. R. China

**Keywords:** Mitogen-activated protein (MAP) kinase, Phylogenetic analysis, Signal molecules, Stress, qRT-PCR, TRV-VIGS, Cotton

## Abstract

**Background:**

Mitogen-activated protein kinase (MAPK) cascades play a crucial role in plant growth and development as well as biotic and abiotic stress responses. Knowledge about the MAPK gene family in cotton is limited, and systematic investigation of MAPK family proteins has not been reported.

**Results:**

By performing a bioinformatics homology search, we identified 28 putative MAPK genes in the *Gossypium raimondii* genome. These MAPK members were anchored onto 11 chromosomes in *G. raimondii*, with uneven distribution. Phylogenetic analysis showed that the MAPK candidates could be classified into the four known A, B, C and D groups, with more MAPKs containing the TEY phosphorylation site (18 members) than the TDY motif (10 members). Furthermore, 21 cDNA sequences of MAPKs with complete open reading frames (ORFs) were identified in *G. hirsutum* via PCR-based approaches, including 13 novel MAPKs and eight with homologs reported previously in tetraploid cotton. The expression patterns of 23 MAPK genes reveal their important roles in diverse functions in cotton, in both various developmental stages of vegetative and reproductive growth and in the stress response. Using a reverse genetics approach based on tobacco rattle virus-induced gene silencing (TRV-VIGS), we further verified that *MPK9*, *MPK13* and *MPK25* confer resistance to defoliating isolates of *Verticillium dahliae* in cotton. Silencing of *MPK9*, *MPK13* and *MPK25* can significantly enhance cotton susceptibility to this pathogen.

**Conclusions:**

This study presents a comprehensive identification of 28 mitogen-activated protein kinase genes in *G. raimondii*. Their phylogenetic relationships, transcript expression patterns and responses to various stressors were verified. This study provides the first systematic analysis of MAPKs in cotton, improving our understanding of defense responses in general and laying the foundation for future crop improvement using MAPKs.

**Electronic supplementary material:**

The online version of this article (doi:10.1186/s12870-014-0345-9) contains supplementary material, which is available to authorized users.

## Background

Stressors including salinity, limited water availability, extreme temperatures and fungal pathogens severely limit crop productivity [1]. Cotton is the world’s most important natural textile fiber and a significant oilseed crop. Four cultivated cotton species have been domesticated independently, including the tetraploids *G. hirsutum* L. (AD)_1_ and *G. barbadense* L. (AD)_2_, and diploids *G. herbaceum* L. (A_1_) and *G. arboreum* L. (A_2_). Among these, allotetraploid Upland cotton has significant advantages including high yield potential and adaptability to diverse environments, accounting for >95% of worldwide cotton production (National Cotton Council, 2012, http://www.cotton.org.econ/cropinfo/index.cfm). One of the major ways to sustain increases in cotton production in many regions of the world affected by abiotic and biotic stresses involves mining key genes for stress tolerance improvement. Protein phosphorylation and dephosphorylation are major defense mechanisms for controlling cellular functions in response to external signals. The mitogen-activated protein kinase (MAPK) cascade is one of the universal signaling pathways involved in responses to external stimuli [2-6]. MAPK cascades are composed of three sequentially activated kinase, i.e., MAP kinase kinase kinase (MAPKKK), MAP kinase kinase (MAPKK) and MAP kinase (MAPK) [7]. MAPKs are a specific class of serine/threonine protein kinases. As the last component of the MAPKKK-MAPKK-MAPK cascade, MAPK plays crucial roles in signal transduction of extracellular stimuli in eukaryotes by phosphorylating various downstream targets [8-10].

According to amino acid sequencing, MAPK contains 11 domains (I–XI) that are necessary for the catalytic function of serine/threonine protein kinase, and domains VII and VIII of MAPKs are well conserved [11]. MAPKs carry either a Thr-Glu-Tyr (TEY) or Thr-Asp-Tyr (TDY) phosphorylation motif at the active site, which can be classified into four major groups (A, B, C and D) based on the presence of TDY and TEY motifs [12].

Recently, a number of studies employing molecular and biochemical approaches have revealed that plant MAPKs play an important role in responses to a broad variety of biotic and abiotic stresses including wounding, pathogen infection, temperature, drought and salinity stress as well as plant hormones [5,13,14]. Utilizing genome-wide scans, the MAPK gene family has been systematically investigated in *Arabidopsis* [12], tomato [15], tobacco [16], wheat [17], rice [18] and soybean [19]. In *Arabidopsis*, *MPK3*, *MPK4* and *MPK6* are involved in stress responses, and both *MPK3* and *MPK6* are dependent on salicylic acid signaling [7]. In addition, *MPK4* and *MPK6* in *Arabidopsis* are also related to the cold stress response [20]. Several studies on MAPKs have been reported in cotton. *GhMPK2* and *GbMPK3* are upregulated by diverse abiotic stresses and likely play a role in drought and oxidative stress tolerance [21,22]. *GhMPK6* plays an important role in abscisic acid -induced catalase1 expression and H_2_O_2_ production [23], while *GhMPK6a* negatively regulates osmotic stress and bacterial infection [24]. Two additional MAPKs, *GhMPK7* and *GhMPK16*, are involved in plant defense responses and the regulation of certain components of multiple stress-signaling pathways [25,26]. Nevertheless, our knowledge of the MAPK gene family in cotton is limited.

The completion of the genome-sequencing project for *G. raimondii* has made it possible for the first time to identify MAPK family members in *Gossypium* species on a genome-wide scale. In this study, we identified 28 putative MAPK genes in the *G. raimondii* genome and analyzed their sequence phylogeny, genomic structure, chromosomal location and adaptive evolution. Our data, combined with sequence data from *G. raimondii* (http://www.phytozome.net) and ESTs from different cotton species in the NCBI databases (http://www.ncbi.nlm.nih.gov/dbEST/)*,* led to the identification of 21 cDNA sequences of MAPKs with complete ORFs in *G. hirsutum* via PCR-based approaches, including 13 novel MAPKs and eight with homologs reported previously in tetraploid cotton. We investigated the temporal and spatial expression profiles of MAPK genes in different tissues and in response to different hormone, temperature and stress treatments in tetraploid cultivated cotton species. Furthermore, we verified the functional roles of three MAPKs that are significantly induced by *Verticillium dahlia* in response to cotton *V. dahliae* resistance. This study opens up the possibility of exploring the use of MAPKs to improve stress tolerance in future cotton-breeding programs.

## Results

### Genome-wide identification of MAPK genes and their chromosomal distribution

To identify MAPK genes from *G. raimondii*, HMMER software version 3.0 [27] and the Pfam protein families database with the MAPK domain (PF00069) [28] were used to screen the *G. raimondii* genomic database (http://www.phytozome.net) [29]. Furthermore, we used 20 *Arabidopsis* MAPK protein sequences as direct queries to screen the potential MAPKs. These predicted GrMAPK sequences were confirmed by FGENESH (http://www.softberry.com/berry.phtml) and the conserved protein domains in their sequences were analyzed by ExPASy proteomics Server (http://www.expasy.ch/prosite/) [30]. After extensive bioinformatics analysis of the *G. raimondii* genome databases, a total of 28 MAPK genes were identified. In addition, we anchored expressed sequence tag (EST) sequences for four cotton species, *Gossypium hirsutum* (Gh), *G. barbadense* (Gb), *G. arboreum* (Ga) and *G. raimondii* (Gr), which we downloaded from the GenBank EST database (http://www.ncbi.nlm.nih.gov/dbEST/). We found that 611 ESTs, including 68 from *G. raimondii*, 422 from *G. hirsutum*, 51 from *G. barbadense* and 70 from *G. arboreum* matched these MAPK members with at least one EST hit (e ≤ −10). These MAPK genes were predicted to encode proteins 366 to 628 amino acids in length, with putative molecular weights ranging from 42.35 to 71.5 KDa and pIs ranging from 5.13 to 9.32.

**Figure 1:**
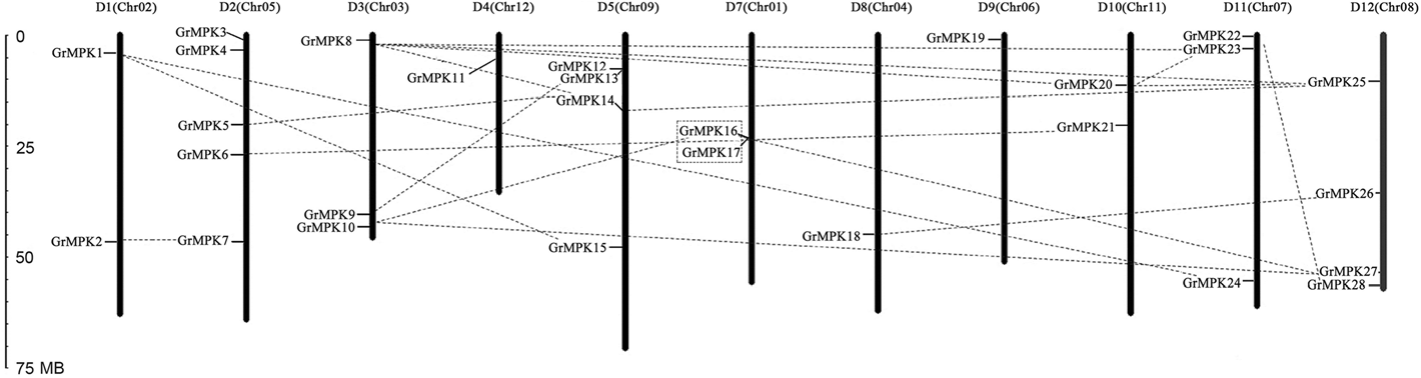
**Chromosomal distribution of MAPK genes in** ***G. raimondii.*** The chromosome numbers are indicated at the top of each bar. The chromosome numbers from D1 to D5, and D7 to D12 were consistent with our newly-updated interspecific genetic map in allotetraploid cultivated cotton species reported recently (Zhao *et al*. [32]), and the scaffolds name from *G. raimindii* genome was showed in the bracket. Lines were drawn to connect duplicated genes. The nomaclature of MAPKs were based on the order of the chromosomes in *G. raimondii*.

To elucidate the chromosomal distribution of these MAPK genes, we integrated 13 scaffolds of the *G. raimondii* genome (named Chr01 to Chr13) from Paterson *et al.* [31] with a previously reported high-density interspecific genetic map of allotetraploid cultivated cotton species [32]. The collinearity between the genetic map and the cotton D_5_ genome revealed homologs between 13 Dt chromosomes in tetraploid cotton species and 13 scaffolds of *G. raimondii*. We reordered the 13 scaffolds of *G. raimondii* according to the corresponding D1 to D13 chromosomes in tetraploid cotton species [32]. As a result, 28 candidate MAPK genes were matched to 11 scaffolds of the D_5_ genome, except for corresponding chromosomes D6 and D13. We designated *MPK1* to *MPK28* based on the order of the homologs on chromosomes (Figure 1). The chromosomal distribution pattern of these MAPK genes is non-random. For example, five MAPKs are found on D2, while four MAPKs each are found on D5 and D12. The remaining members are also localized to different chromosomes: three MAPKs each are present on D3 and D11; two MAPKs each are present on D1, D7 and D10; only one MAPK is present on D4, D8 and D9, respectively. Information about the MAPK genes, including their gene names, origins, chromosome locations, isoelectric points (pIs), molecular weights (MWs) and subcellular localizations, are shown in Additional file 1: Table S1.

### Classification, structure and variation of MAPK genes in *Gossypium raimondii*

**Figure 2:**
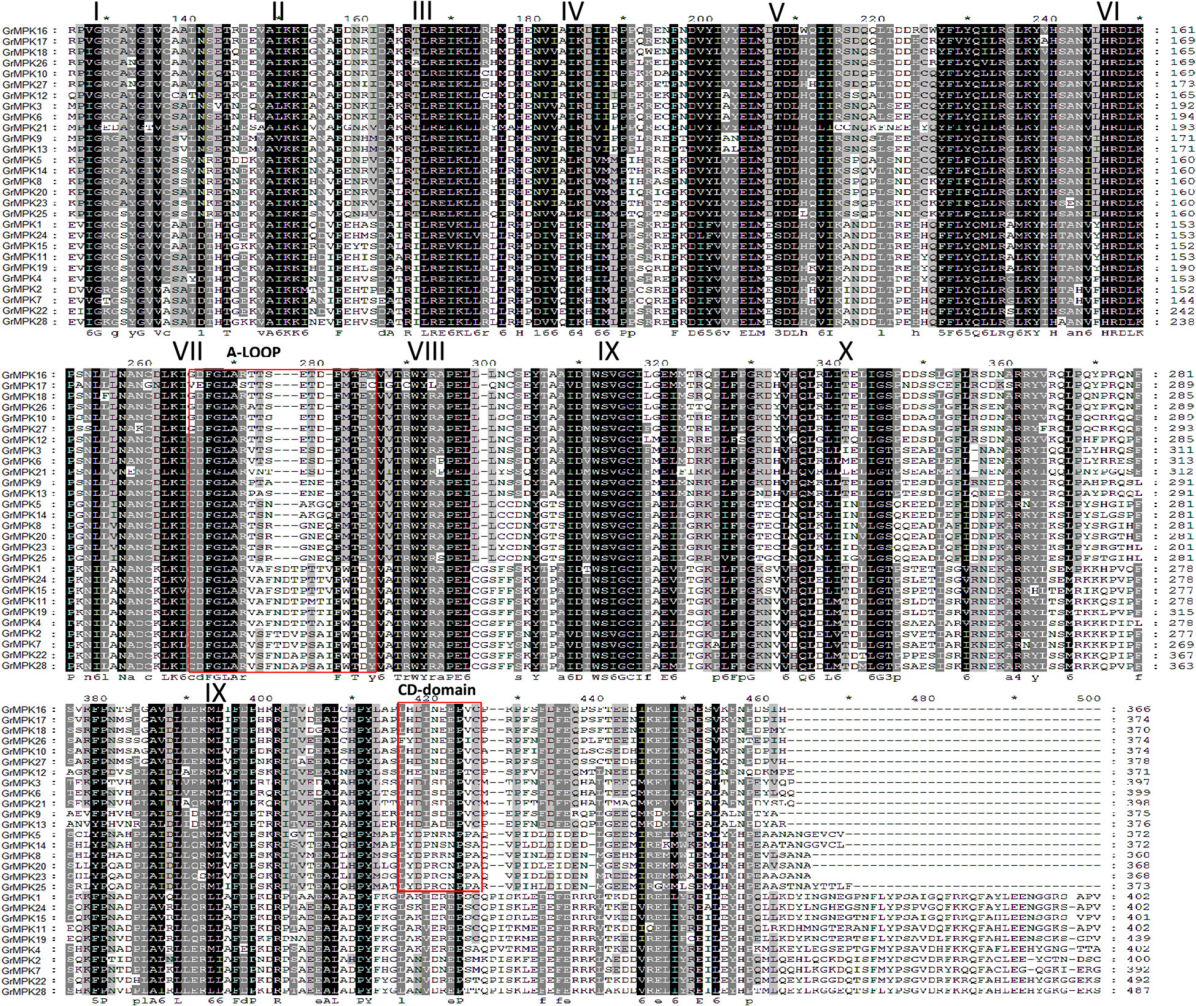
**Comparison of the amino acid sequences of GrMAPKs.** Roman numerals indicate regions containing the 11 domains (I–XI) found in the cotton PK subdomains. The A-Loop, CD-domain and phosphorylation-activation motif (TEY and TDY) are indicated with red boxes.

Alignment of GrMAPK amino acid sequences revealed that all of the GrMAPK proteins contain 11 domains (I–XI; Figure 2). TEY or TDY motifs of GrMAPKs are located in the activation loop between kinase subdomain VII and VIII. All GrMAPK protein sequences contain four types of special subdomains, including the active site, ATP binding site, substrate-binding site and activation loop (A-loop). Phylogenetic analysis indicated that GrMAPK could be divided into four major groups (A, B, C and D), with five members in group A, seven in group B, six in group C and 10 in group D. GrMAPKs in subgroup A, B, C possess a Thy-Glu-Tyr (TEY) and a short C-terminus containing a common docking (CD) domain consisting of the sequence [LHY]Dxx[DE]EpxC, whereas those of subgroup D possess a Thr-Asp-Tyr (TDY) activation domain, without a CD domain but with a relatively long C-terminal region.

**Figure 3:**
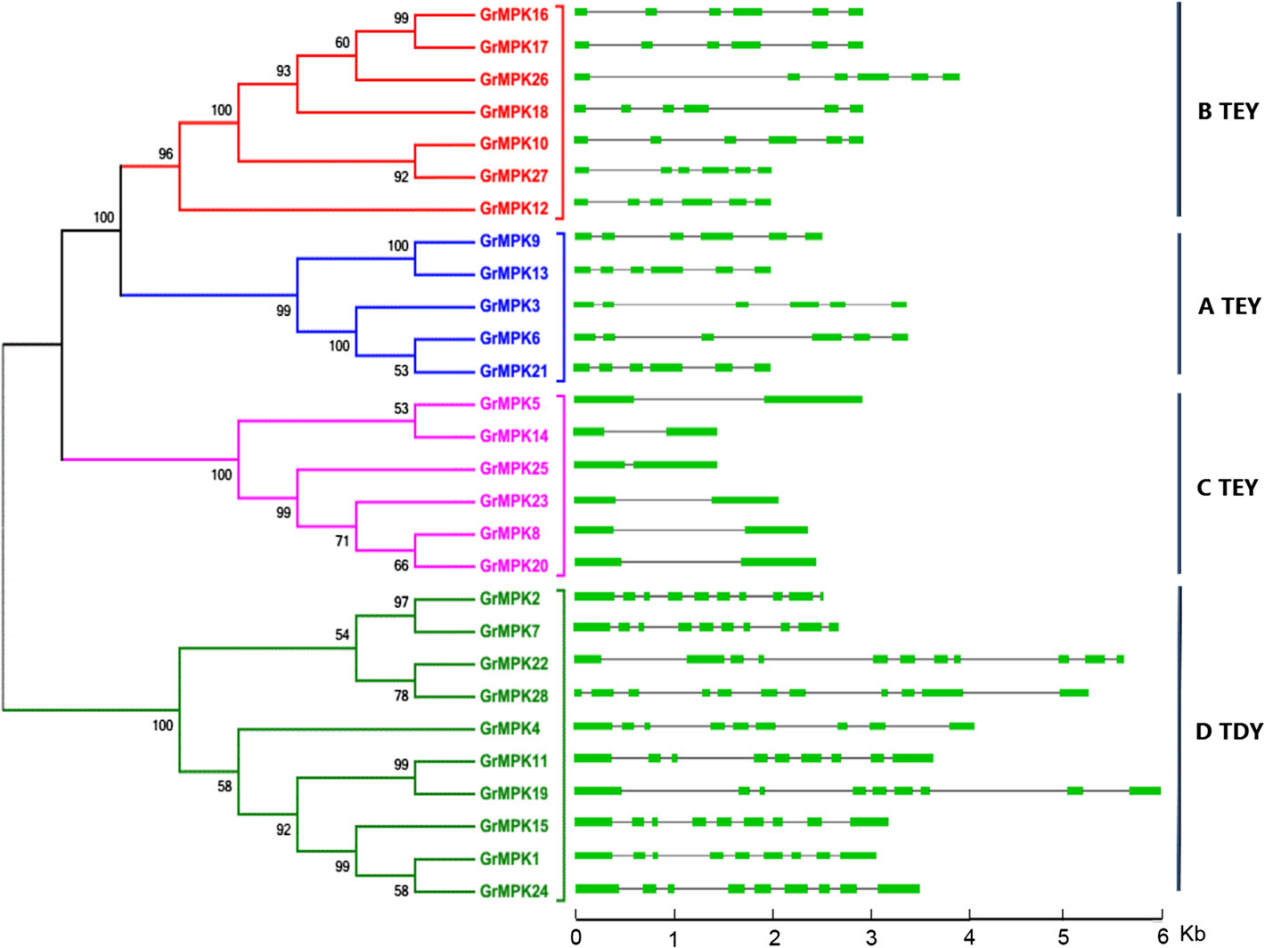
**Intron and exon organization of** ***G. raimondii*** **MAPK genes (** ***GrMPKs*** **).** Introns and exons are represented by black lines and colored boxes, respectively. *GrMPKs* were grouped according to phylogenetic classification. Phylogenetic analysis was done using the ML method with 1,000 resampling replicates. Bootstrap values (%) based on 1000 replicates are indicated beside the nodes.

Analysis of exon/intron structures further revealed the classification of the GrMAPK family (Figure 3). *GrMAPKs* in groups A and B exhibit a highly conserved distribution of exons and introns consisting of six exons of conserved length and five introns of variable sizes. Each MAPK in group C contains only two similarly sized exons, except that *GrMPK25* has a shorter intron. Compared with these three highly conserved groups, MAPKs in group D show a complex distribution of exons and introns; *GrMPK2* and *GrMPK7* have 10 exons, *GrMPK22* and *GrMPK28* have 11 exons, while the others are composed of nine exons.

The phylogenetic relationships of MAPK genes have been systematically investigated in *Arabidopsis* [12], tomato [15], tobacco [16], wheat [17], rice [18] and soybean [19]. Here, to examine the evolutionary relationships of MAPK members in *G. raimondii* and other species, 20 MAPK genes in *Arabidopsis*, 38 in *G. max*, 17 in *O. sative* and 28 in *G. raimondii* were individually selected to construct an unrooted tree based on the alignment of the full MAPK amino acid sequences using the Maximum likelihood method via MEGA5.1 [33]. The information for MAPK genes from different species was showed in Additional file 2: Table S2.

**Figure 4:**
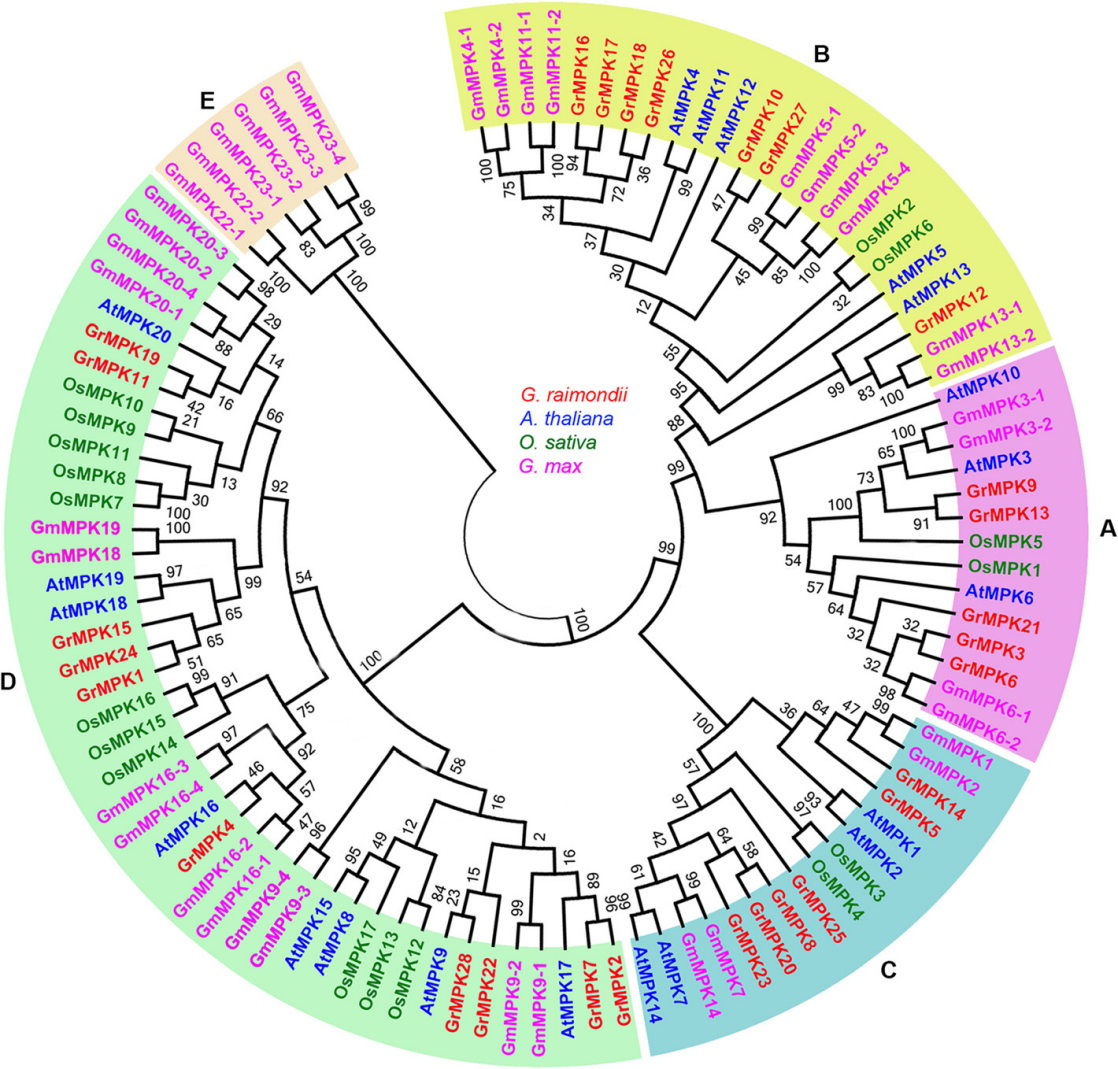
**Phylogenetic relationships of MAPK family genes from** ***G. raimondii*** **,** ***A. thaliana*** **,** ***O. sativa*** **, and** ***G. max.*** Amino acid sequences were aligned using ClustalX software and subjected to phylogenetic analysis using the ML method with 1,000 resampling replicates. Bootstrap values (%) based on 1000 replicates are indicated beside the nodes. GrMAPKs are highlighted in red and the other MAPKs from *A. thaliana*, *O. sativa* and *G. max* are shown in different colors.

Phylogenetic analysis indicated that all of the MAPKs could be classified into the A, B, C, D and E groups (Figure 4). Interestingly, more MAPK members from *Arabidopsis*, *G. max* and *G. raimondii* contain the TEY phosphorylation site than the TDY motif. There are 12 AtMAPKs, 18 GmMAPKs and 18 GrMAPKs containing the TEY motif, whereas eight AtMAPKs, 14 GmMAPKs and 10 GrMAPKs belong to the TDY groups, with an exception of six GmMAPKs containing the TQY motif. By contrast, the rice genome contains more MAPKs with the TDY phosphorylation site than the TEY motif; 11 OsMAPKs have the TDY motif but only seven contain the TEY motif. These results indicate that MAPKs containing the TEY motif might play more important roles in dicot plants than MAPKs containing the TDY motif. The orthlogous relationship among MAPK genes in *G. raimondii*, *Arabidopsis*, *O. sativa* and *G. max* was showed in Additional file 3: Table S3.

Recent studies have shown that the *G. raimondii* genome has undergone at least two rounds of genome-wide duplication [29]. To understand the expansion mechanism of the *G. raimondii* MAPK gene family, we investigated tandem and segmental duplication events of MAPK gene family members on the 11 chromosomes by genome synteny analysis. As shown in Figure 1, 19 paralogs in 28 *G. raimondii* MAPKs were identified, including 18 segmental duplication events between chromosomes and one tandem duplication event within the same chromosome (*GrMPK16* and *GrMPK17*). Furthermore, these paralogs are clustered together in the phylogenic tree and share similar exon-intron structures. These results indicate that segmental duplication events have played a significant role in MAPK gene expansion in the *G. raimondii* genome.

### Cloning and expression analysis of MAPK genes in *G. hirsutum* acc. TM-1

Based on predicted sequence information, we performed PCR cloning of MAPK genes by designing gene-specific primers (Additional file 4: Table S4) and amplifying the transcripts of given tissues of *G. hirsutum* acc. TM-1. We ultimately obtained 21 MAPK cDNA sequences with complete ORFs (GenBank accession Nos: KM190106-KM190126), including 13 novel MAPKs and eight with homologs that had been reported previously, with seven in Upland cotton and one in Sea Island cotton (Additional file 1: Table S1). Other seven genes with partial cDNA sequences were also identified.

**Figure 5:**
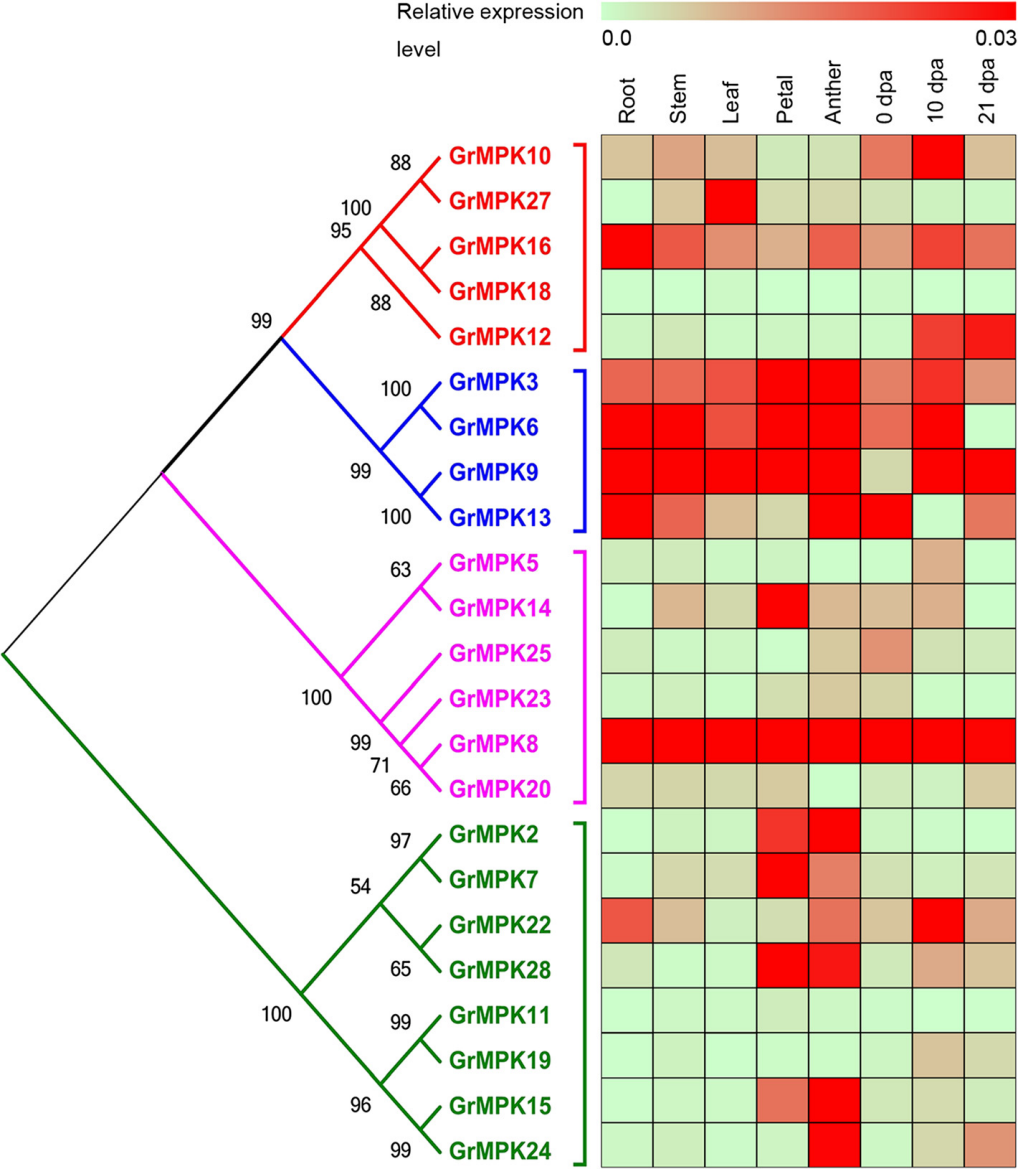
**Real-time qRT-PCR analysis of MAPK genes in different tissues and organs in** ***G. hirsutum*** **acc. TM-1.** A total of eight cotton tissues (root; stem; leaf; petal; anther; ovule at 0 day post anthesis (DPA); fiber at 10 DPA; and fiber at 21 DPA) were sampled to analyze. Differences in gene expression intensities are shown in colors indicated in the scale. Phylogenetic analysis was done using the ML method with 1,000 resampling replicates. Bootstrap values (%) based on 1000 replicates are indicated beside the nodes.

To explore the possible physiological functions of MAPKs, we designed gene-specific qRT-PCR primers (Additional file 4: Table S4) to elucidate the expression levels of MAPK genes in tetraploid cotton. In total, we detected the expression patterns of 23 MAPK genes in different tissues and organs of *G. hirsutum* acc. TM-1, including roots, stems, leaves, petals, anthers, ovules and fibers at three different developmental stages (0 days post-anthesis [dpa], 10 dpa and 21 dpa). As shown in Figure 5, MAPKs from different groups showed diverse expression patterns in different tissues and organs, with partial overlap observed in a range of physiological processes. In detail, expression pattern of individual gene for each tissue/organ tested was showed in Additional file 5: Figure S1.

First, five genes, including *MPK3*, *MPK6*, *MPK9* and *MPK13* in group A and *MPK8* in group C, were predominantly expressed in both vegetative and reproductive organs, with the highest expression observed for *MPK8* in all tissues and organs examined. Second, *MPK16* and *MPK27* (in group B) showed preferential expression in vegetative organs; *MPK16* was ubiquitously expressed in all organs and preferentially expressed in roots, while *MPK27* showed that the highest expression levels in leaf tissues, with 10-fold higher expression in leaves than in other organs. Third, nine genes were predominantly expressed in reproductive organs. Of these, four genes, including *MPK10* and *MPK12* in group B and *MPK22* and *MPK24* in group D, had the highest expression levels in fiber tissues, and five genes, including *MPK14* in group C and *MPK2*, *MPK7*, *MPK15* and *MPK28* in group D, were preferentially expressed in anthers, petals or both. Two additional genes, *MPK5* and *MPK25* (in group C) were expressed moderately in reproductive organs, with preferential expression in fibers at different developmental stages. Fourth, five genes, i.e., *MPK18* in group B, *MPK20* and *MPK23* in group C, and *MPK11* and *MPK19* in group D, showed very low levels of expression in all tested tissues and organs. These results indicate that MAPK genes from the same or different groups showed differential but overlapping expression patterns in different tissues, suggesting that genes belonging to the same group may have diverse functions, whereas MAPK genes from different groups may share the same function.

### Expression profiles of MAPKs in response to various stress-related signals

**Figure 6:**
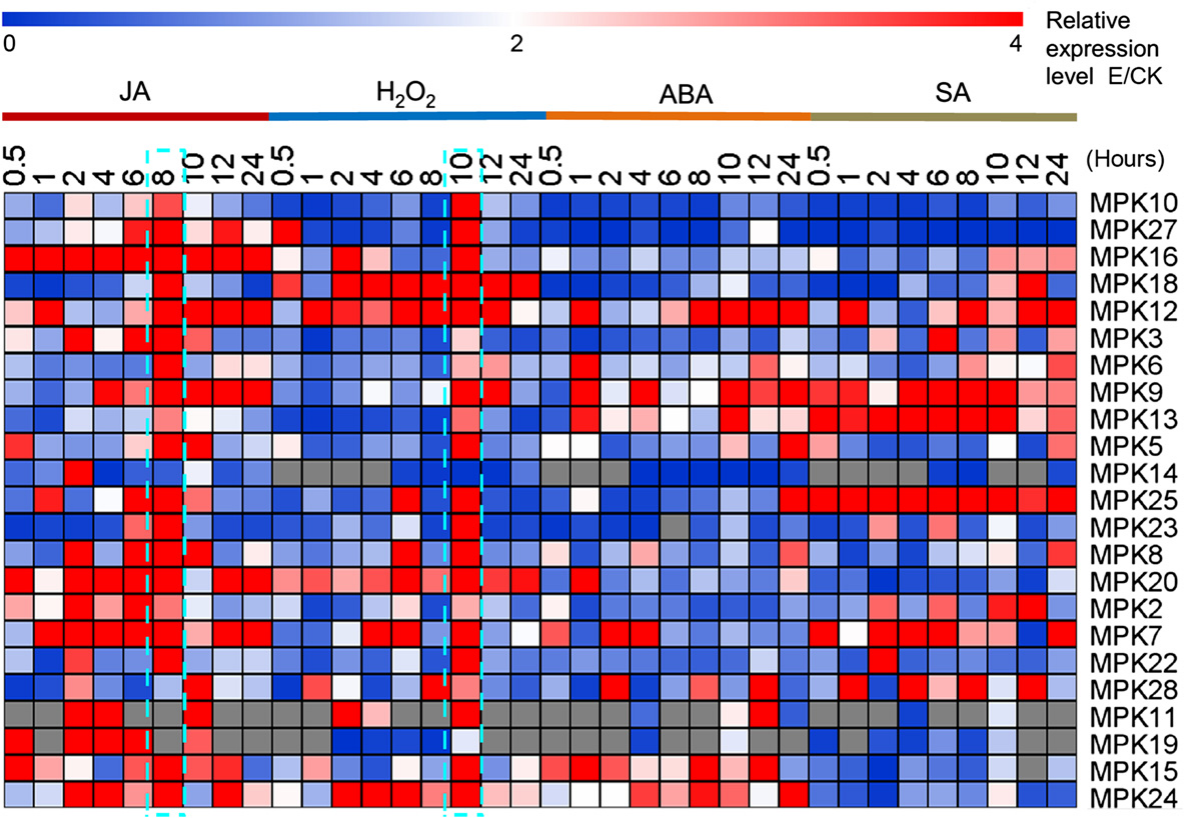
**Relative expression of** ***G. hirsutum*** **MAPK genes under stress-related signal treatments.** The data are presented in clusters using the fold-change (E/C) of relative expression for all MAPK genes in response to stress-siganl treatments (Experiment), in comparison to their respective controls (Control). Red and blue colors represent increased or decreased expression levels, respectively, in comparison to controls. The stress-related signals included JA, H_2_O_2_, ABA and SA, respectively.

To investigate the roles of MAPK genes under various stress-related stimuli, we performed qRT-PCR to detect the differences in their expression abundance after exposure to three stress-related signaling compounds (abscisic acid [ABA], salicylic acid [SA], jasmonic acid [JA]) or an oxidative stress inducer [H_2_O_2_]). A total of 23 of the MAPKs were induced by at least one of four inducers, implying that MAPKs play important roles in signaling pathways. Among these, ten were simultaneously induced and accumulated at higher levels after all four treatments; ten were induced by three inducers; one gene by two inducers and two genes by only one of the four inducers (Figure 6). For further details, expression pattern of individual gene under each treatment was showed in Additional file 6: Figure S2.

Under JA conditions, 23 MAPKs were induced significantly. However, MAPKs from the four groups showed differently altered expression patterns. Transcript levels of genes in group A and B and most in group C significantly increased, reaching a peak at 8 h after treatment, while those of *MPK14* and *MPK20* (in group C) significantly increased and reached two peaks at 2 h and 4 h, respectively. The expression levels of the other MAPK genes in group D significantly increased, quickly reaching a peak at different time points.

Twenty one MAPK genes were significantly upregulated after H_2_O_2_ treatment. In addition, *MPK6* was induced, and its expression reached two peak values at 8 and 12 h, respectively. The other genes were significantly upregulated, reaching their highest levels at 10 h of treatment, including three genes in group A, five in group B, five in group C, and seven in group D. Fifteen MAPK genes, including three in group A, two in group B, four in group C, and six in group D, were significantly upregulated after ABA treatment, with diverse expression patterns. Finally, fifteen MAPK genes were significantly upregulated after SA treatment. Of these, six MAPKs were induced, including four in group A, one each in group C and D, with a peak observed at 6 or 8 h, while three genes each in group B, C, and D reached peak values at other time points.

### Expression profiles of MAPKs in response to abiotic stress

**Figure 7:**
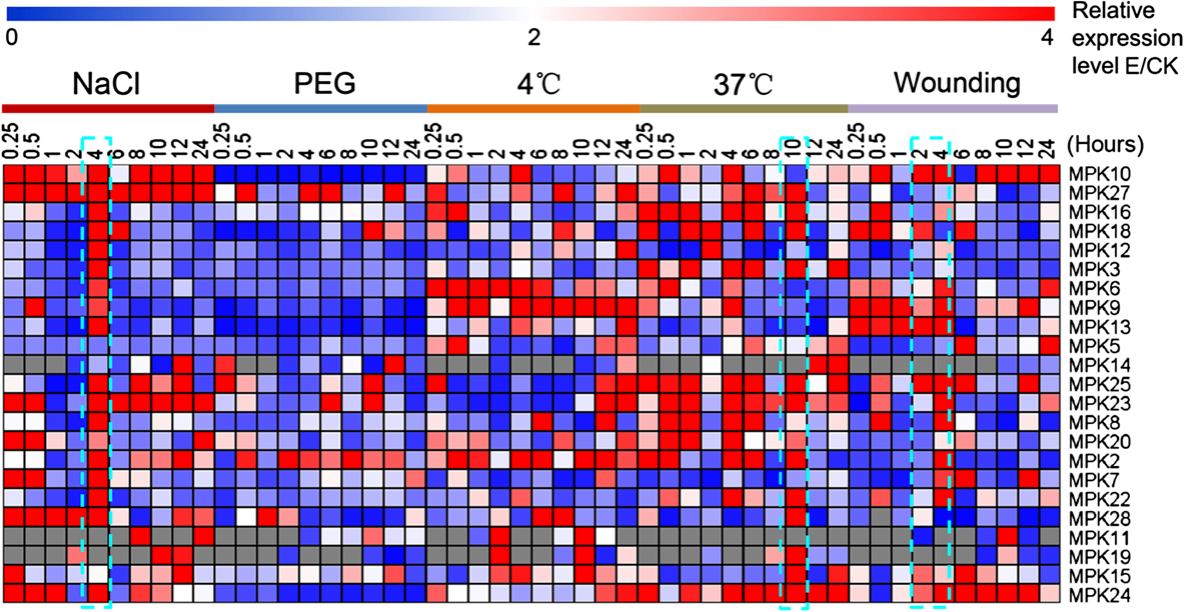
**Relative expression of** ***G. hirsutum*** **MAPK genes under different stress treatments.** The data are presented in clusters using the fold-change (E/C) of relative expression for all MAPK genes in response to different treatments (Experiment), in comparison to their respective controls (Control). Red and blue colors represent increased or decreased expression levels, respectively, in comparison to controls. The stressors included NaCl, PEG, 4°C, 37°C, and wounding treatment, respectively.

To investigate the roles of MAPK genes under various abiotic stress conditions, we performed qRT-PCR to detect the differences in their expression after five stress treatments (salinity, drought, cold, heat and wounding). As shown in Figure 7, the transcript levels of 22 MAPK genes significantly increased after NaCl treatment. In addition to *MPK5*, *MPK9* and *MPK14*, MAPK genes in groups A, B and C were induced and accumulated at 4 h. All members of group D were also induced, but their expression patterns were diverse. The detailed information for expression pattern of individual genes under each treatment was showed in Additional file 7: Figure S3.

Eleven MAPK genes were significantly induced under drought treatment, including two in group B, four in group C, and five in group D. In addition, 23 MAPK genes were induced and highly expressed after low temperature treatment (4°C), with diverse expression pattern. Except for *MPK3*, all of the MAPK genes were induced and expressed at high levels. Moreover, 21 MAPK genes were induced and highly expressed upon exposure to high temperature conditions. Of these, two genes each in groups B and C and five in group D were significantly upregulated and reached a peak at 10 h after treatment, while other twelve genes were induced and reached peak values at other time points. Finally, 21 MAPK genes were induced and upregulated when the seedling leaves were cut with scissors. Of these, three in group A, five each in groups B and C, and eight in group D were significantly induced and reached peak values at different time points.

In total, the 23 detected MAPK genes were widely induced by all types of abiotic stress (Table 1). Among these genes, eight were induced and expressed at higher levels under all five abiotic stress treatments. Thirteen and two MAPK genes were induced by four and three abiotic stresses, respectively. These expression patterns suggest that MAPK genes carry out multiple physiological functions to help the plant adapt to various complex environmental challenges.

**Table 1:** **Expression profiles of MAPK genes under different stress treatments in cotton**

**Gene**	**Group**	**Signalling molecules**				**Environmental stress factors**				
		**JA (100uM)**	**H**_**2**_ **O**_**2**_ **(10 mM)**	**ABA (100uM)**	**SA (10 mM)**	**Salt (200 mM)**	**PEG6000 (20%)**	**4°C**	**37°C**	**Wounding**
*MPK10*	Group B	**	**	D	D	**	D	**	**	**
*MPK27*	Group B	**	**	**	D	**	**	**	**	**
*MPK16*	Group B	**	**	*	**	**	*	**	**	**
*MPK18*	Group B	**	**	-	**	**	**	**	**	**
*MPK12*	Group B	**	**	**	**	**	D	**	**	**
*MPK3*	Group A	**	**	*	**	**	-	**	**	*
*MPK6*	Group A	**	**	**	**	**	D	**	**	**
*MPK9*	Group A	**	**	**	**	**	D	**	**	**
*MPK13*	Group A	**	**	**	**	**	D	**	**	**
*MPK5*	Group C	**	**	**	**	-	-	**	**	**
*MPK14*	Group C	**	D	D	D	**	**	**	**	D
*MPK25*	Group C	**	**	**	**	**	**	**	**	**
*MPK23*	Group C	**	**	*	**	**	**	**	**	**
*MPK8*	Group C	**	**	**	**	**	*	**	**	**
*MPK20*	Group C	**	**	**	*	**	**	**	**	**
*MPK2*	Group D	**	**	**	**	**	**	**	**	**
*MPK7*	Group D	**	**	**	**	**	**	**	*	**
*MPK22*	Group D	**	**	*	**	**	-	**	**	**
*MPK28*	Group D	**	**	**	**	**	**	**	**	**
*MPK11*	Group D	**	**	**	*	**	**	**	/	**
*MPK19*	Group D	**	*	*	D	**	D	**	**	**
*MPK15*	Group D	**	**	**	*	**	**	**	**	**
*MPK24*	Group D	**	**	**	*	**	D	**	**	**

Note: For hormone treatments, the leaves of seedlings were harvested at 0, 0.5, 1, 2, 4, 6, 8, 10, 12 and 24 h after treatment;

For the environmental stress factor treatments, the leaves of seedlings were harvested at 0, 0.25, 0.5, 1, 2, 4, 6, 8, 10, 12 and 24 h after treatment;

“**” and “*” indicate significant difference at P < 0.01 and P < 0.05, respectively;

“-” represents no change and weak upregulation; “D” represents significant reduction in MAPK gene expression after treatment;

“/” represents absent data. The Student’s t-test was performed between treated samples and untreated samples.

### Paralogs of MAPKs show diverse expression patterns

To investigate whether these duplicated paralog pairs were with the same expression patterns, we compared their expression profiles in different organs and under different stress treatments (Table 2). In organs, only the correlation coefficient between *MPK8* and *MPK14* was greater than 0.5, indicating a positive correlation and similar expression patterns between these two genes. However, other pairs had no clear positive or negative correlation. Notably, the correlation coefficient between *MPK20* and *MPK25* was lower than −0.5, suggesting distinctly different expression patterns between these two genes. Comparison analysis indicated that paralogs of MAPKs from the same ancestor showed differential expression in different tissues and organs, implying that these genes evolved via gene duplication followed by expressional divergence.

**Table 2:** **Pearson correlation coefficients of the expression profiles of paralogous pairs**

**Gene1**	**Gene2**	**Similarity**	Correlation coefficient*	**Correlation coefficient**	**Correlation coefficient**
			**(organs)**	**(hormone)**	**(abiotic stress)**
*MPK2*	*MPK7*	86.73%	0.24	0.40	0.26
*MPK5*	*MPK14*	93.28%	−0.04	**0.63**	0.27
*MPK8*	*MPK14*	84.51%	**0.86**	**0.60**	0.10
*MPK8*	*MPK20*	93.48%	0.34	**0.51**	**0.56**
*MPK8*	*MPK23*	91.85%	0.15	**0.89**	**0.84**
*MPK8*	*MPK25*	85.87%	−0.39	0.26	**0.69**
*MPK9*	*MPK13*	92.27%	−0.46	**0.71**	**0.73**
*MPK10*	*MPK27*	90.37%	−0.15	**0.83**	−0.03
*MPK10*	*MPK16*	86.63%	0.03	**0.66**	−0.06
*MPK14*	*MPK25*	80.65%	−0.30	0.14	0.07
*MPK16*	*MPK27*	84.96%	−0.26	**0.83**	**0.64**
*MPK20*	*MPK23*	92.39%	−0.49	0.33	**0.67**
*MPK20*	*MPK25*	86.96%	**−0.61**	−0.06	**0.53**
*MPK22*	*MPK28*	77.28%	−0.09	−0.07	0.23

*Correlation coefficient: r > 0.5: positive correlation, showed in bold type; 0 < r < 0.5: no clear positive correlation; −0.5 < r < 0: no clear negative correlation; r < −0.5: negative correlation.

Furthermore, correlation analysis indicated that there were eight paralogs involved in stress-related signals and seven in abiotic stress with values greater than 0.5, implying positively correlated expression between paralogs under stress. Unlike *MPK2-MPK7*, *MPK14-MPK25* and *MPK22-MPK28*, four paralogs, i.e., *MPK8-MPK20*, *MPK8-MPK23*, *MPK9-MPK13* and *MPK16-MPK27*, showed clear positive correlations under both stress-related signal and abiotic stress treatment, and other seven paralogs showed positive correlations under one or two stress conditions. Taken together, these results suggest that MAPKs may have retained functional conservation after gene duplication to help plants cope with different stresses, acting as the main contributors to wide adaptation during the cotton evolutionary process.

### Potential functional roles of three MAPK genes in *Verticillium dahliae* resistance, as determined by TRV-VIGS

**Figure 8:**
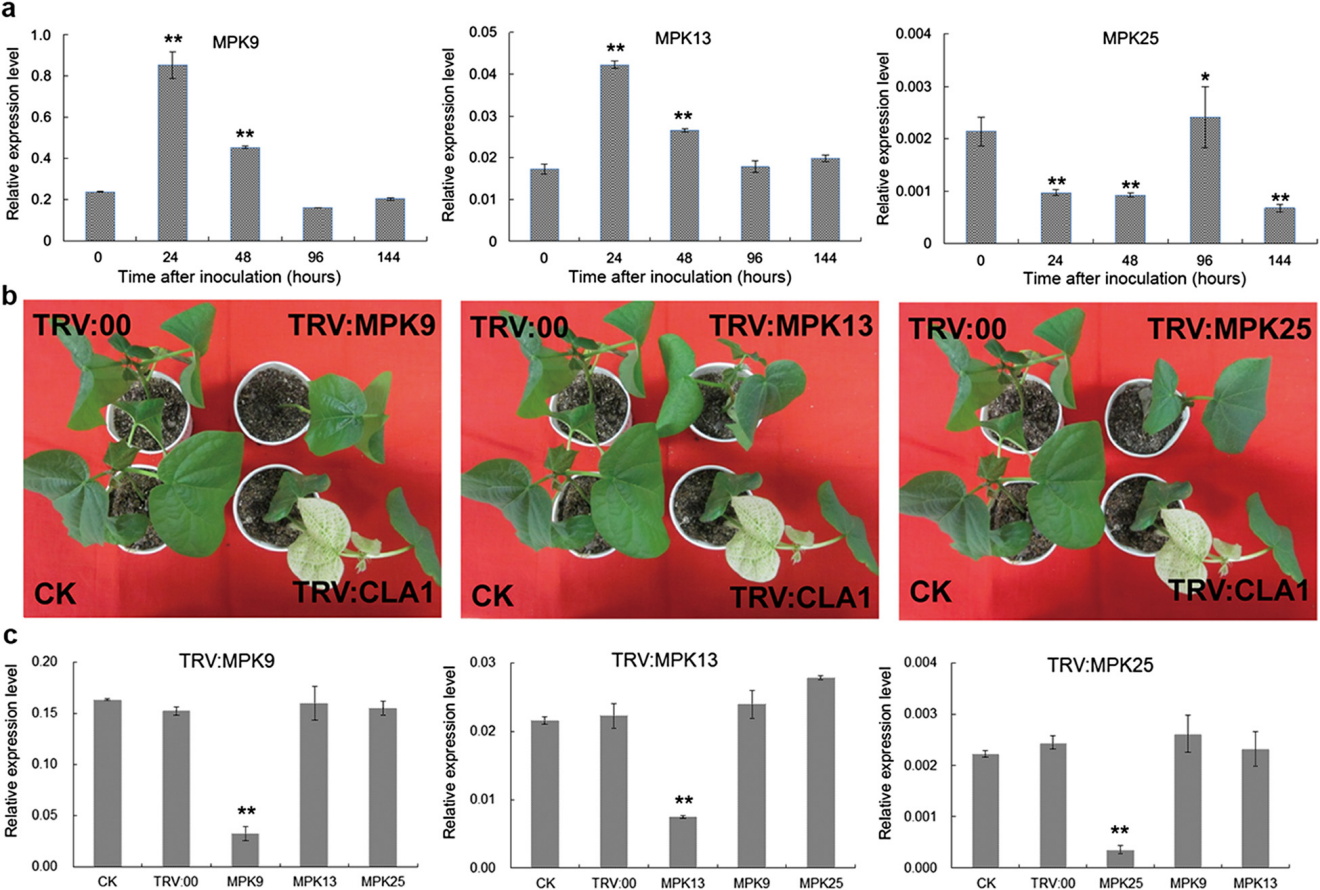
**Expression patterns of three MAPK genes induced by** ***V. dahliae*** **and VIGS analysis. (a)** Q-PCR analysis of the expression of three MAPK genes after inoculation by *V. dahliae*. The error bars were calculated based on three biological replicates using standard deviation. “*”: significant difference (P < 0.05); “**”: significant difference (p < 0.01). **(b)** Phenotypes after TRV-VIGS silencing of three MAPK genes and *GhCLA1*. After two weeks post-treatment with TRV1 and TRV2/TRV2-MPKs, the treated plants exhibited normal growth. TRV1- and TRV-GhCLA1-treated plants exhibited a photobleaching phenotype. **(c)** Gene expression of MPK9, MPK13 and MPK25 in silenced and control plant leaves by Q-PCR analysis; The error bars were calculated based on three biological replicates using standard deviation. “*”: significant difference (P < 0.05); “**”: significant difference (p < 0.01). The cotton histone 3 (AF026714) was used as the reference gene.

Three MAPKs, including *MPK9*, *MPK13* and *MPK25*, were significantly induced after *Verticillium dahliae* inoculation (Figure 8a). The transcript levels of *MPK9* and *MPK13* significantly increased, with the highest peak observed at 24 h of treatment. *MPK25* was significantly downregulated in response to inoculation after 24 h and 48 h, and its expression recovered to high levels at 96 h post-inoculation.

Virus-induced gene silencing (VIGS) has been successfully used in cotton [34-36]. To further investigate the function of *MPK9*, *MPK13* and *MPK25* in *V. dahliae* resistance, we constructed recombinant viruses to silence endogenous genes in cotton, producing constructs TRV2:*MPK9*, TRV2:*MPK13* and TRV2:*MPK25*, with TRV1-TRV2 for the mock treatment. To validate the reliability of VIGS in cotton, we silenced an indicator gene, *CLA1* (CLOROPLASTOS ALTERADOS 1, encoding 1-deoxy-D-xylulose-5- phosphate synthase), producing plants with a photobleached phenotype. At least 15 plants were infiltrated per construct at 8 days post-emergence, and untreated plants were grown in the same environment without syringe treatment. Two weeks later, all treated individuals infiltrated with TRV2-*CLA1* showed highly uniform bleaching in newly emerged leaves (Figure 8b). Real-time quantitative PCR confirmed that untreated and mock-treated plants showed the same and high expression levels of *MPK9*, *MPK13* and *MPK25*. However, the transcripts of these three genes exhibited strong silencing in infiltrated TRV2:*MPK9*, TRV2:*MPK13* and TRV2: *MPK25* plants (P < 0.01)(Figure 8c).

**Figure 9:**
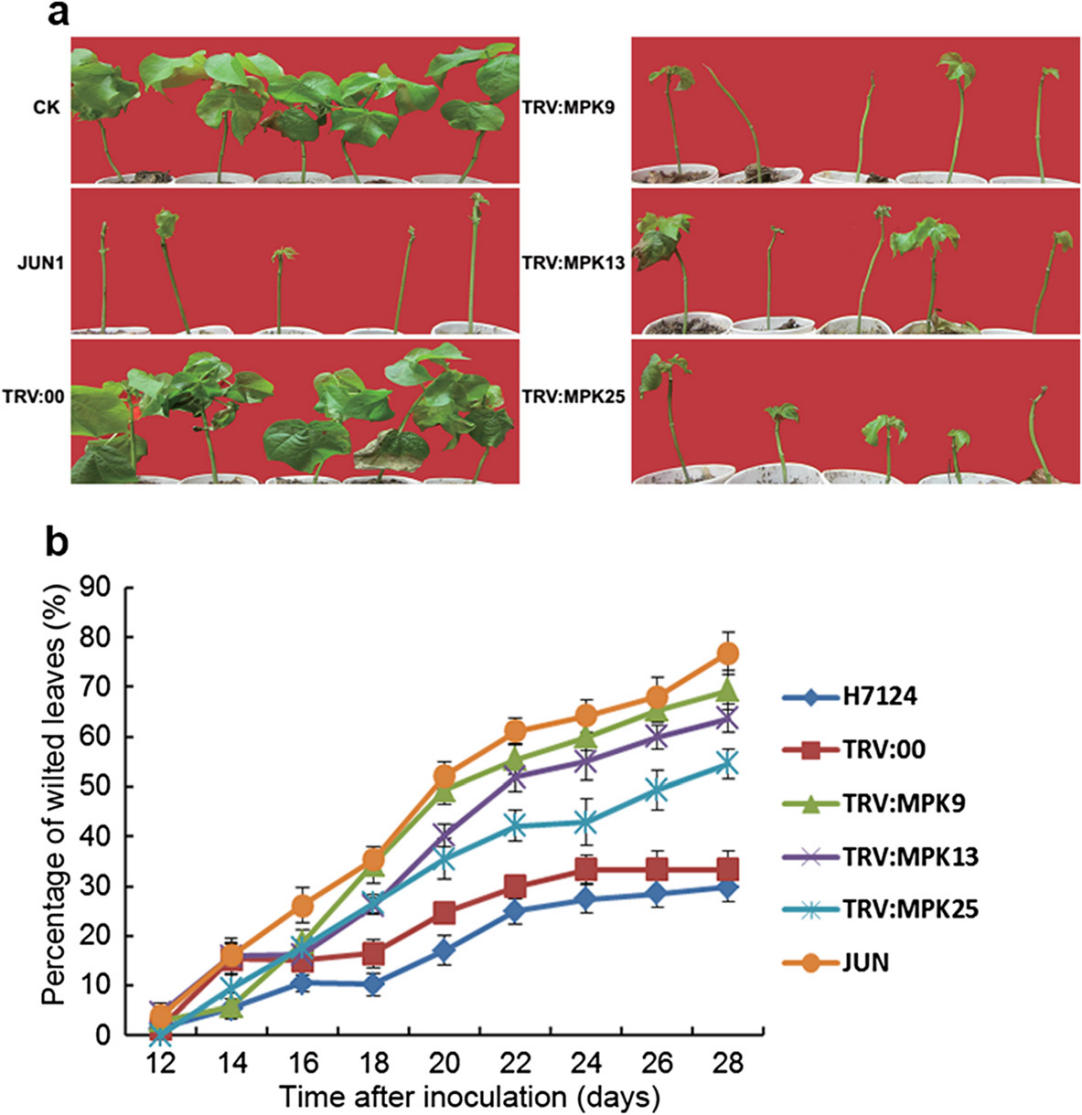
**Silencing of** ***MPK9*** **,** ***MPK13*** **and** ***MPK25*** **enhances plant susceptibility to** ***Verticillium dahlia*** **infection.** Individual genes of the cotton cultivar Hai7124 were first silenced by VIGS, and the plants were then inoculated with *V. dahliae* suspension spores at a concentration of 1 × 10^7^/mL. **(a)** Plant phenotypes at 28 days after *V. dahlia* inoculation. **(b)** Percentage of diseased leaves after *V. dahlia* inoculation. The percentage of diseased leaves was scored. The experiments were repeated using 15 plants per treatment. The error bars were calculated based on three biological replicates using standard deviation.

We inoculated cotton seedlings using dip-infection with liquid containing 1 × 10^7^ *V. dahliae* spores. Two weeks later, spontaneous lesions in stems and yellow leaf veins were found in target gene-silenced plants. Four weeks later, the true leaves of diseased plants exhibited wilting (Figure 9a). In general, the control plants seldom exhibited leaf wilting, with average diseased leaf: healthy leaf ratios of approximately 30%. However, 69.3% of the *MPK9*-silenced plants were severely infected by *V. dahlia*, which was similar to the results observed in susceptible control plants (*G. hirsutum* cv. Junmian 1, with the percentage of diseased plants at 76.8%). Furthermore, 63.75% of the *MPK13*-silenced plants showed a severe wilting phenotype, and 54% of the *MPK25*-silenced plants exhibited wilting symptoms on leaves when infected with *V. dahlia* (Figure 9b). These results demonstrate that silencing of *MPK9*, *MPK13* and *MPK25* compromises the resistance of cotton to this pathogen, and gene-silenced plants exhibited more wilting and etiolated leaves than the vector control plants with P < 0.01 significance. In summary, *MPK9*, *MPK13* and *MPK25* are important components of resistance to *V. dahlia* infection in cotton.

## Discussion

### Characterization of MAPKs in *G. raimondii* and evolution of MAPK genes

Based on the genome scans of several plant genomes, MAPK family genes have been systematically investigated in *Arabidopsis* [12], tomato [15], tobacco [16], wheat [17], rice [18] and soybean [19]. In the current study, a total of 28 MAPKs from *G. raimondii* were identified. These MAPKs were classified into four groups (A, B, C and D) according to their phylogenetic clades, which were similar to those reported in *Arabidopsis* and *O. sativa* [18,37]. We also found that all MAPK proteins contain 11 domains (I–XI; Figure 1), and TEY or TDY motifs of MAPKs are located in the activation loop between kinase subdomain VII and VIII, as described previously [12,38]. The subgroup of A, B and C possesses a Thr-Glu-Tyr (TEY) domain and a short C-terminus containing a common docking (CD) domain that consists of the sequence[LHY]Dxx[DE]EpxC, whereas those of subgroup D possess a Thr-Asp-Tyr(TDY) activation domain, without the CD domain but with a relatively long C-terminal region, which is also consistent with previous reports [39,40]. Previous studies, such as reports in *Arabidopsis*, tobacco, tomato and rice, focused on TEY MAPKs [2]. Interestingly, *Arabidopsis*, *G. max* and *G. raimondii* contain more MAPKs with the TEY phosphorylation site than the TDY motif. By contrast, the *O. sativa* genome contains more MAPKs with the TDY phosphorylation site than the TEY motif. We propose that in dicot plants, MAPKs with the TEY motif play more important roles than MAPKs with the TDY motif.

Phylogenetic analysis between cotton and other plants revealed that GrMAPKs in A, B and C might come from the same ancestor, while D MPAK genes might be paralogous products. The distribution of GrMPAKs is nonrandom, which is similar to the result of a previous study [41]. Based on phylogenetic tree analysis, we found that a large number of MAPKs belong to subgroup D, which is similar to reports in *Arabidopsis*, rice and poplar [12,18,41,42]. These results support the previous suggestion that subgroup D expanded before and after the monocot/dicot split [41]. Comparison of exon-intron structures indicated that the A, B and C groups share a similar number of exons and the lengths of exons are more conserved than those of introns. However, members in the D group have more exons and the lengths of these exons and introns are diverse. Synteny analysis of the *G. raimondii* genome indicated that the MAPK family mainly resulted from segmental duplication.

### Expression patterns of MAPKs imply their functional divergence during plant development and growth

Previous studies have demonstrated that some MAPK genes exhibit tissue specificity in various plants such as *Arabidopsis*, tobacco, poplar, *Brachypodium distachyon*, wheat and *Brassica* [17,37,43-46]. *RsMPK2* was detected in vegetative and reproductive organs, with different expression patterns [47]. *TaMAPK13* is expressed in all tissue, but *TaMPK15* is only expressed at low levels in flowers [17].

In the current study, we observed differential expression patterns of MAPKs in vegetative organs (roots, stems and leaves) and reproductive organs (anthers, petals and fiber tissues at different developmental stages). Five genes were constitutively expressed at high levels in both vegetative and reproductive organs. Two genes were expressed at higher levels in vegetative organs, and the remaining genes were expressed at higher levels in reproductive organs. A recent study demonstrated that *AtMPK4* plays an important role in meiotic cytokinesis during pollen development [48]. *PsMPK3* is involved in fruit set, which is activated by gibberellins and cytokinins [49]. *SlMPK3* is expressed at markedly high levels in stamens [15]. The tissue- or organ-specific MAPK expression patterns observed in the current study indicate their functional divergence during plant development and growth. Interestingly, four MAPK genes, *MPK3*, *MPK6*, *MPK9*, and *MPK13* in Group A and *MPK8* in Group C with higher expression in almost all tested tissues were worth to be further studied for actual function.

### Varied expression of MAPKs in response to stress-related signals and abiotic stressors

Abiotic and biotic stresses such as cold, drought and pathogens seriously affect cotton growth and yield, and studies have focused on the molecular mechanisms underlying the response to these stresses in cotton. To date, an increasing number of studies have shown that MAPKs can regulate plant development, growth and responses to abiotic/biotic stress. In cotton, *GhMPK2* and *GbMPK3* are upregulated by diverse abiotic stresses and likely play a role in drought and oxidative stress tolerance [21,22]. *GhMPK6* plays an important role in ABA-induced CAT1 expression and H_2_O_2_ production [23], whereas *GhMPK6a* negatively regulates responses to osmotic stress and bacterial infection [24]. *GhMPK7* and *GhMPK16* are involved in plant defense responses and the regulation of certain components of multiple stress-signaling pathways [25,26]. Extensive studies have revealed that MAPKs are not only involved in abiotic stress and biotic responses but also in plant development and hormonal signaling. Vlot and coworkers (2009) suggested that SA can regulate responses to biotrophic pathogens and systemic acquired resistance, while JA mediates responses to necrotrophs [50]. Recent studies have shown that ABA is involved in salinity and drought responses [51]. H_2_O_2_ can induce oxidative bursts or the accumulation of reactive oxygen species (ROS) in plant cells. ROS may contribute to resistance by directly killing the invading pathogen or activating cell wall crosslinking and lignification and subsequently strengthening the cell wall to help confine pathogen infection [52].

Systematic analyses of the expression patterns of MAPK genes under stress-related signal treatment showed that ten of 23 (43.5%) MAPK genes were induced by four inducers. Ten (43.5%) and one (4.3%) of these genes were induced by three and two inducers, respectively. Two (8.7%) genes were induced by only one of the four inducers. Accumulating evidence has shown that systemic defense responses in plants are controlled by the mutually antagonistic hormones JA and SA. Our data indicate that all MAPK genes were induced under JA treatment, with 15 were simultaneously induced by SA. This finding implies that these genes are coregulated by JA and SA and highlights the notion that MAPK genes might play key roles in plant defense responses. Furthermore, the gene expression patterns under abiotic stress show that eight of 23 (34.8%) MAPK genes were upregulated by five abiotic stressors. Thirteen (56.5%) and two (8.7%) were upregulated by four or three stressors, respectively. The present results further demonstrate that MAPKs are involved in the response to environmental stress in cotton.

Previous reports have shown that each hormone signaling pathway contributes to an interactive network that coordinates responses to different stresses [53]. In addition, abiotic stress-regulated genes act either in an ABA-dependent or ABA-independent manner, SA can regulate responses to biotrophic pathogens and systemic acquired resistance. Here, among 15 ABA-regulated MAPK genes, 10 were also regulated by SA. Further, our data indicate that eight MAPK genes were upregulated by five stressors and also induced by JA and H_2_O_2_. Among these, five genes were simultaneously induced by four stress-related signals. The widespread induction of MAPK genes in response to diverse stressors and hormones suggests that MAPK genes play a significant role in hormone signaling pathways during stress tolerance.

Gene duplication is followed by functional diversification. A comparison of the expression patterns of paralogous genes demonstrated that most, but not all, of these genes showed similar responses towards various hormone and abiotic treatments. For instance, *MPK9-MPK13*, *MPK16-MPK27*, *MPK8-MPK20* and *MPK8-MPK23* paralogs showed similar responses to both hormone and abiotic treatments. The paralogs *MPK5-MPK14*, *MPK8-MPK14*, *MPK10-MPK16* and *MPK10-MPK27* showed similar expression patterns in response to hormones, and the paralogs *MPK8-MPK25, MPK20-MPK23* and *MPK20-MPK25* showed similar expression patterns in response to abiotic stresses. Our results suggest that these pairs of MAPKs share similar functions under abiotic and/or hormone treatment, respectively. An examination of the overall transcription patterns suggests that gene duplication resulted in partially overlapping functions; the redundant functions of MAPK genes may be beneficial for protecting the cell from various stress conditions. On the other hand, paralogs of MAPK genes also showed an interesting pattern of functional divergence in different organs and tissues, implying that MAPK genes may play a crucial role in driving evolutionary novelty and adaptation to new environments.

### *MPK9*, *MPK13* and *MPK25* are required for resistance against *Verticillium dahliae* in cotton

Gene expression patterns are usually an indicator of gene functions. In the current study, we found that MAPK genes were generally responsive to biotic and abiotic stress treatments, suggesting that they play important roles in responses to environmental stress and pathogens. *Verticillium wilt* is a serious disease that significantly affects the yield and quality of cotton.

Accumulating evidence demonstrates that the MAPK cascade plays an important role in the regulation of pathogen-induced defenses. *AtMPK3/AtMPK6* are activated by pathogens and regulate the pathogen defense response pathway, and *AtMPK3/AtMPK6* are involved in abiotic stress response (salt, drought, cold, wounding) and hormone signal pathways [54,55]. *OsMPK5* is also induced by abiotic stresses, pathogen infection and ABA treatment [56]. In cotton, *GhMPK6a*, *GhMPK7* and *GhMPK16* are involved in the pathogen-resistance response [24-26]. Here, we found that *MPK9*, *MPK13* and *MPK25* were significantly upregulated in cotton roots after inoculation with *V. dahliae,* and these three genes were upregulated in leaves after exposure to JA, H_2_O_2_, ABA and SA. We speculate that *MPK9*, *MPK13* and *MPK25* are involved in regulating the pathogen response. Using VIGS technology [34-36], we further investigated the function of *MPK9*, *MPK13* and *MPK25* in *V. dahliae* resistance. Statistical analysis showed that silencing of whether *MPK9*, *MPK13* or *MPK25* by VIGS increased significantly the susceptibility of cotton to *V. dahliae.* Compared with the two other genes, plants harboring a silenced *MPK9* gene were more severely infected by *V. dahlia*, implying that *MPK9* plays an important role in *V. dahlia* resistance in cotton.

## Conclusions

A total of 28 GrMAPKs were identified based on the genome sequence of *G. raimondii*, and 21 cDNA sequences of MAPKs with complete ORFs were cloned from *G. hirsutum*. Phylogenetic tree and motif analysis showed that GrMAPKs could be classified into four groups, comparable to those in *Arabidopsis*, *O. sativa* and *G. max.* Most MAPKs showed different temporal and spatial expression patterns in vegetative and reproductive organs, and crosstalk occurred under biotic/abiotic stress and stress-related signal treatment. VIGS analysis indicated that *MPK9*, *MPK13* and *MPK25* are important components in cotton resistance to *V. dahliae* infection. Our work provides a reference for systematically elucidating the important roles of MAPKs in cotton growth, development and responses to abiotic and biotic stresses and for effectively utilizing MAPKs in cotton stress tolerance breeding.

## Methods

### Prediction, mapping and analysis of the MAPK gene family

Genes and proteins annotated in *G. raimondii* were downloaded from http://www.phytozome.net. HMMER software version 3.0 [27] and the Pfam protein family database with the MAPK domain (PF00069) [28] were used as a query to screen the *G. raimondii* genomic database. Expressed sequence tag (EST) sequences for four cotton species, *Gossypium hirsutum*(Gh), *G. barbadense* (Gb), *G. arboreum* (Ga) and *G. raimondii* (Gr), were downloaded from the GenBank EST database (http://www.ncbi.nlm.nih.gov/dbEST/).

Mapping of MAPK genes was performed using MapInspect (http://www.plantbreeding.wur.nl/UK/software_mapinspect.html). The exon/intron structures of individual GrMAPK genes were determined by aligning the cDNA sequences to their corresponding genomic DNA sequences.

### Conserved domain detection and subcellular location predication

The programs INTERPROSCAN, SMART, MOTIF and PLANTSP were employed to detect conserved domains. If a given protein sequence contained the MAP Kinase signature, PK domain and ATP-binding domain, it was regarded as a candidate member of the cotton MAPK family. The subcellular localization of each GrMAPK was analyzed using CELLO v2.5 server (http://cello.life.nctu.edu.tw/) [57].

### Sequence alignments and phylogenetic construction

Multiple sequence alignments of the MAPK domain with 28 amino acids were performed using ClustalX (ver.1.83) [58], and a phylogenetic tree was constructed by the Maximum likelihood (ML) method in MEGA 5.1 (www.megasoftware.net) [33]; the bootstrap test of phylogeny was performed with 1,000 replications. In addition, the amino acid sequences of MAPKs from four plants (*Arabidopsis*, *O. sativa*, *G. max* and *G. raimondii*) were initially aligned and used to construct phylogenetic trees.

### Plant materials and treatments

*G. hirsutum* L. acc TM-1, a genetic standard line of Upland cotton, was used for tissue/organ expression analysis. The plants were cultivated under normal field conditions. Petals and anthers were sampled on the day of flowering, and ovules and fibers were excised from developing flower buds or bolls on selected days post anthesis (dpa). Roots, stems and leaves were collected from two-week-old seedlings. The materials were quick-frozen in liquid nitrogen and stored at −70°C before use.

*G. hirsutum* L. cv. Jinmian 19, which exhibits high tolerance to abiotic stress, was used for the abiotic stress treatments. Cotton seedlings (*G. hirsutum* L. cv. Jinmian 19) were grown in a growth chamber under greenhouse conditions at 28°C under a 16 h light/8 h dark cycle. Three-week-old cotton seedlings were used for the following treatments. For signaling substance treatments, leaves were sprayed with 100 μM JA, 100 μM ABA, 100 mM SA or 10 mM H_2_O_2_ (ddH_2_O as a solvent control). For the salt and drought treatments, the roots of cotton seedlings were irrigated with 200 mM NaCl and 20% PEG, respectively (ddH_2_O as a mock control). For temperature stress treatments, the seedlings were placed in a growth chamber at a high temperature (37°C) or a low temperature (4°C; 28°C as a mock). Seedling leaves were cut with scissors for wound treatment. The leaves were harvested at the appropriate time points as indicated (triplicate samples were collected at each time point [n = 3 seedlings]), frozen in liquid nitrogen and stored at −70°C for further analysis.

*G. barbadense* L. cv. Hai7124, which exhibits *Verticillium* resistance, was used for fungal pathogen (*V. dahliae*) inoculation. For pathogen treatment, the roots of Hai7124 seedlings were dipped in *V. dahliae* strain V991 conidial suspensions containing 10^7^ spores mL^−1^. The roots were harvested at the appropriate time points, quick-frozen in liquid nitrogen and stored at −70°C before use. *G. hirsutum* L. cv. Junmian 1, which is susceptible to *Verticillium*, was used as a susceptible plant control.

### RNA isolation and real-time PCR analysis

Total RNA was extracted from cotton seedling leaves using the CTAB-acidic phenol extraction method [59]. RNA was then treated with DNase I (Invitrogen, http://www.invitrogen.com/) to remove genomic DNA, and 2 μg of total RNA was used for first-strand cDNA synthesis. The primer pairs used for real-time PCR were designed using Beacon Designer 7.0 according to cotton MAPK gene sequences. The amplified fragment lengths were between 75 bp and 200 bp, and the annealing temperature was between 58°C and 60°C. The cotton *histone3* (AF024716) gene was used as the reference gene [60].

The amplification reactions of real-time PCR were performed on an ABI 7500 Real Time PCR System (Applied Biosystems, USA) using SYBR Green (Bio-Rad, USA) with three replicates. The amplification parameters were as follows: denaturation at 95°C for 10 min, 40 cycles of denaturation at 95°C for 15 s, annealing between 58°C and 60°C for 15 s, extension at 72°C for 15 s. For the melting curve stage, the default settings were chosen. The expression levels of MAPK genes were calculated according to Livak and Schmittgen [61].

### Cloning of MAPK genes from *G. hirsutum* acc. TM-1

Based on the predicted sequences, gene-specific primers were designed to obtain the complete coding sequences. The primer pairs for all genes and the optimal melting temperature are listed in Additional file 4: Table S4; the transcripts from various tissues of *G. hirsutum* acc. TM-1 were used for amplification. Standard PCR reactions were performed using High-fidelity ExTaq DNA Polymerase (TaKaRa Biotechnology [Dalian] Co., Ltd., China). The PCR products were cloned into the pMD18-T Vector (TaKaRa) according to the manufacturer’s instructions and sequenced from plasmid DNA templates. At least six clones per gene were randomly picked and sequenced. The cDNA sequences of the MAPK genes were determined using alignment analysis with their corresponding sequences obtained from bioinformatic analysis.

### Construction of VIGS vectors and agro-infiltration

The pTRV1 and pTRV2 VIGS vectors were kindly provided by Dr. Libo Shan of Texas A&M University (College Station, TX, USA). The constructs contained the following fragments: TRV2:*MPK9*, a 501-bp fragment of an *MPK9* cDNA fragment that corresponds to base positions 47–538 bp; TRV2: *MPK13*, a 391-bp fragment of an *MPK13* cDNA fragment that corresponds to base positions 43–434 bp and TRV2:*MPK25*, a 392-bp fragment of an *MPK25* cDNA fragment that corresponds to base positions 58–440 bp. These fragments were amplified by PCR from TM-1 cDNA using primers with XbaI/XhoI enzyme sites for TRV2:*MPK9* and XbaI/SacI sites for both TRV2:*MPK13* and TRV2:*MPK25*, for insertion into TRV2, respectively. The primer pairs used for the construction of VIGS vectors harboring the three MAPK genes are listed in Additional file 4: Table S4. The control vector pTRV2-*GhCLA1* was the kind gift of Dr. Xinyu Wang, Nanjing Agricultural University.

Plasmids containing TRV1, TRV2, TRV2:*MPK9*, TRV2:*MPK13* and TRV2:*MPK25* were individually introduced into *Agrobacterium tumefaciens* strain GV3101. Agrobacterium cultures carrying the recombinant TRV vectors were grown overnight at 28°C in LB medium containing the antibiotics 50 μg/mL kanamycin and 25 μg/mL rifampicin. The cultures were then inoculated into 50 mL LB medium (50 μg/mL kanamycin, 25 μg/mL rifampicin) at a concentration of 1:100 and cultured, with shaking, to an OD of 0.5 at 28°C. The cells were pelleted by centrifugation at 1,180 × g at room temperature for 5 min and resuspended in infiltration media (10 mM MgCl_2_, 10 mM MES and 200 μM acetosyringone).

The cell suspensions were incubated at room temperature for 3 h and then Agrobacterium GV3101 carrying TRV1 and TRV2:*MPK9/13/25* was infiltrated into two fully expanded cotyledons of eight-day-old cotton seedlings (Hai7124) using a needleless 1 mL syringe at a 1:1 ratio, with Junmian-1 serving as a susceptible control. For mock treatment and the technical control, the same plants were infiltrated with a 1:1 mixture of Agrobacterium carrying TRV1 and TRV2 or TRV1 and TRV2:*CLA1*, respectively. The plants were grown at 23/22°C (day/night) in a growth chamber with a 16 h light/8 h dark cycle for four weeks before they were used for the assays. Untreated plants were grown under the same conditions but were not wounded. VIGS experiments were repeated at least three times with more than 16 plants for each construct per repeat.

### Pathogen inoculation

The defoliating isolate V991 of *V. dahliae* was grown on potato dextrose agar for 4 d at room temperature (25°C) and then incubated in Czapek’s medium at 25°C for 5 d. The spore suspensions were prepared at 1 × 10^7^ conidia mL^−1^ for inoculation of cotton seedlings by dip-infection.

## Additional files

Additional file 1: Table S1. Genome-wide bioinformatic analysis of MAPK genes in *Gossypium.*
Additional file 2: Table S2. The information for MAPK genes from different species used in phylogenetic analysis. Additional file 3: Table S3. The orthlogous relationship among MAPKs in *Arabidopsis*, *O. sativa*, *G. max*, and *G. raimondii*. Additional file 4: Table S4. Oligonucleotide primers used in this study. Additional file 5: Figure S1. Expression patterns of the 23 MAPK genes in various tissues in cotton by quantitative real time PCR analysis. 1: root; 2: stem; 3: leaf; 4: petal; 5: anther; 6: ovule at 0 day post anthesis (DPA); 7: fiber at 10 DPA; 8: fiber at 21 DPA. The Y-axis indicates relative expression levels and the X-axis indicates different tissues. The error bars were calculated based on three biological replicates using standard deviation. Additional file 6: Figure S2. Expression patterns of MAPK genes under stress-related signal treatments (a, JA; b, H_2_O_2_; c, ABA; d, SA). The expression levels data were presented as the mean fold by comparing treated samples with controls. The Y-axis indicates relative expression levels and the X-axis indicates the hours of stress-related signal treatments. The error bars were calculated based on three biological replicates using standard deviation. “*”: significant difference (P < 0.05); “**”: significant difference (p < 0.01). Additional file 7: Figure S3. Expression patterns of MAPK genes under stress treatments (a, NaCl; b, PEG; c, 4°C; d, 37°C; e, wounding). The expression levels data were presented as the mean fold by comparing experiments and controls samples. The Y-axis indicates relative expression levels and the X-axis indicates the hours of stress treatments. The error bars were calculated based on three biological replicates using standard deviation. “*”: significant difference (P < 0.05); “**”: significant difference (p < 0.01).
