# Supplementary figures and images for: Genome-wide identification of mitogen-activated protein kinase gene family in Gossypium raimondii and the function of their corresponding orthologs in tetraploid cultivated cotton
Source: BMC Plant Biol. 2014 Dec 10;14:345. doi: 10.1186/s12870-014-0345-9 (PMC4270029; doi:10.1186/s12870-014-0345-9)

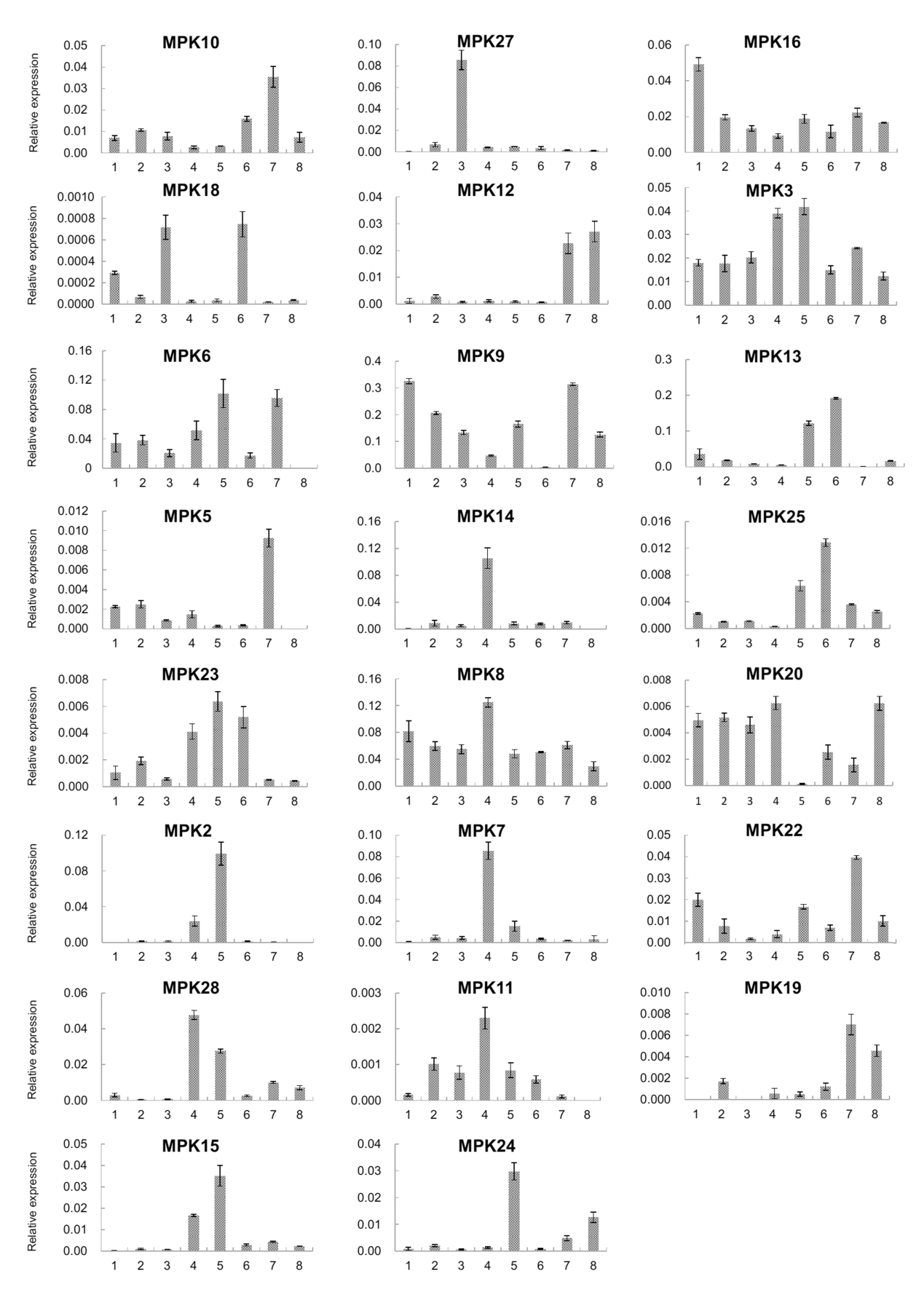

Supplement: Additional file 5: Figure S1. — Expression patterns of the 23 MAPK genes in various tissues in cotton by quantitative real time PCR analysis. 1: root; 2: stem; 3: leaf; 4: petal; 5: anther; 6: ovule at 0 day post anthesis (DPA); 7: fiber at 10 DPA; 8: fiber at 21 DPA. The Y-axis indicates relative expression levels and the X-axis indicates different tissues. The error bars were calculated based on three biological replicates using standard deviation. [file 12870_2014_345_MOESM5_ESM.docx]

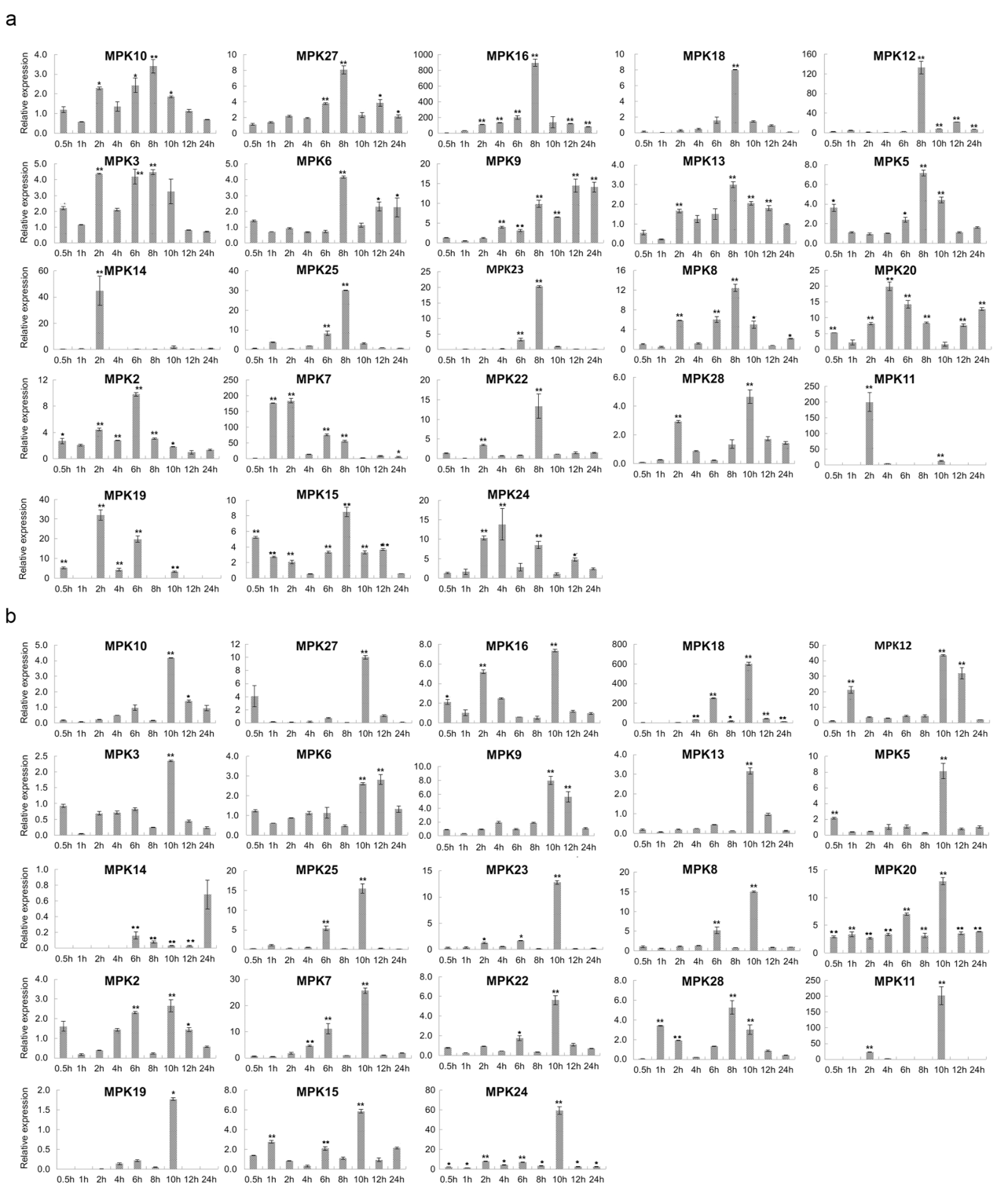


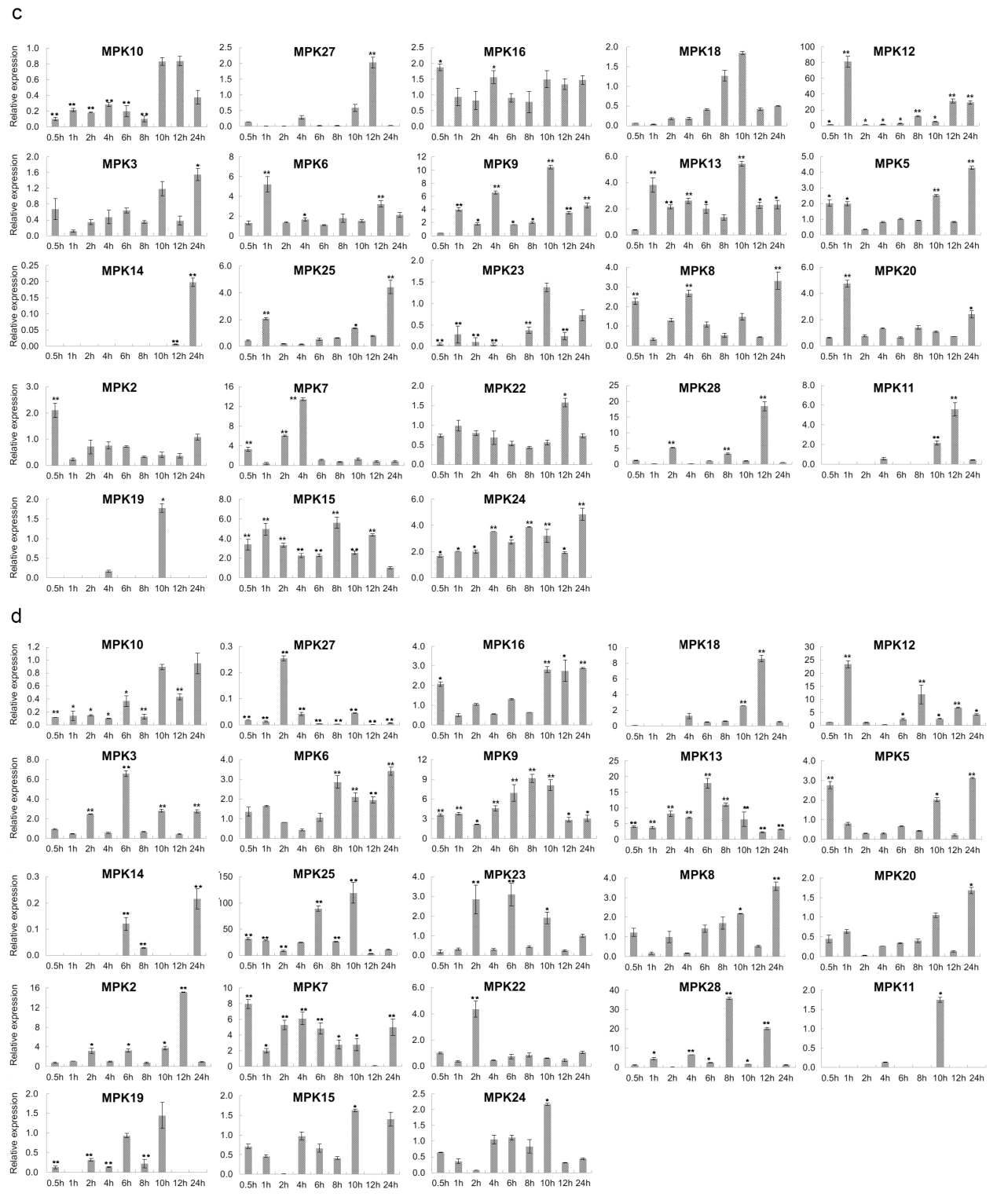

Supplement: Additional file 6: Figure S2. — Expression patterns of MAPK genes under stress-related signal treatments (a, JA; b, H2O2; c, ABA; d, SA). The expression levels data were presented as the mean fold by comparing treated samples with controls. The Y-axis indicates relative expression levels and the X-axis indicates the hours of stress-related signal treatments. The error bars were calculated based on three biological replicates using standard deviation. “*”: significant difference (P < 0.05); “**”: significant difference (p < 0.01). [file 12870_2014_345_MOESM6_ESM.docx]

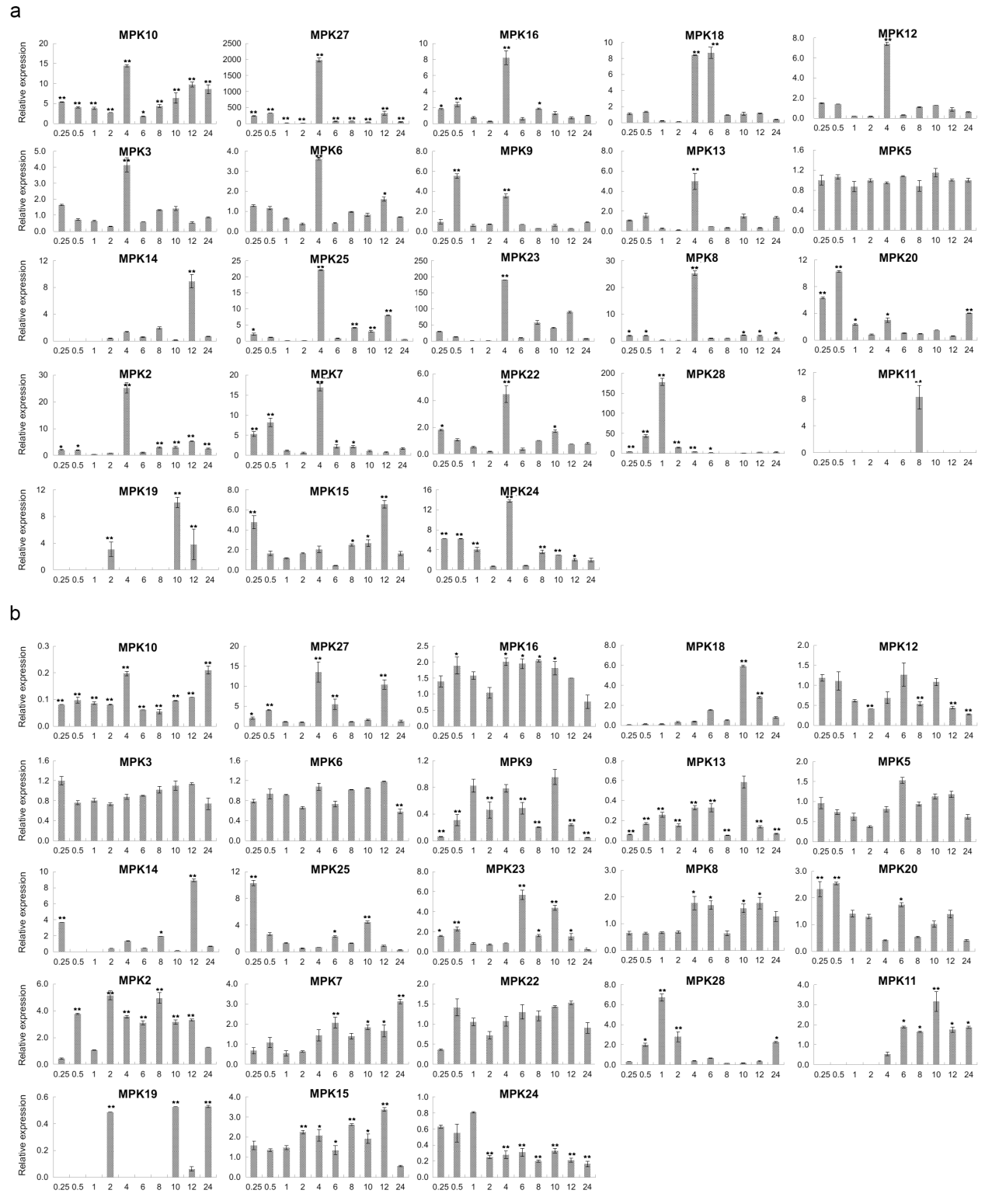


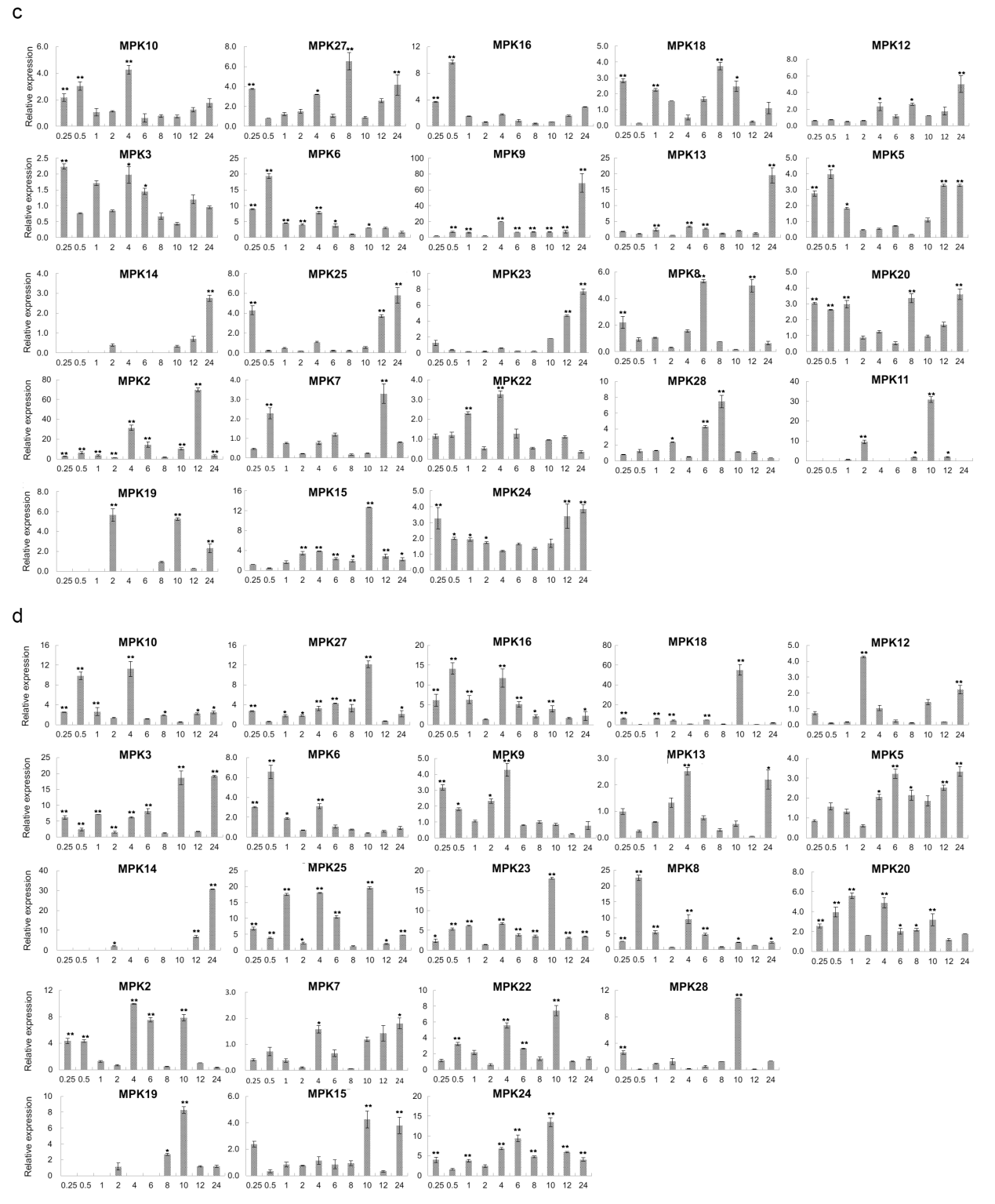


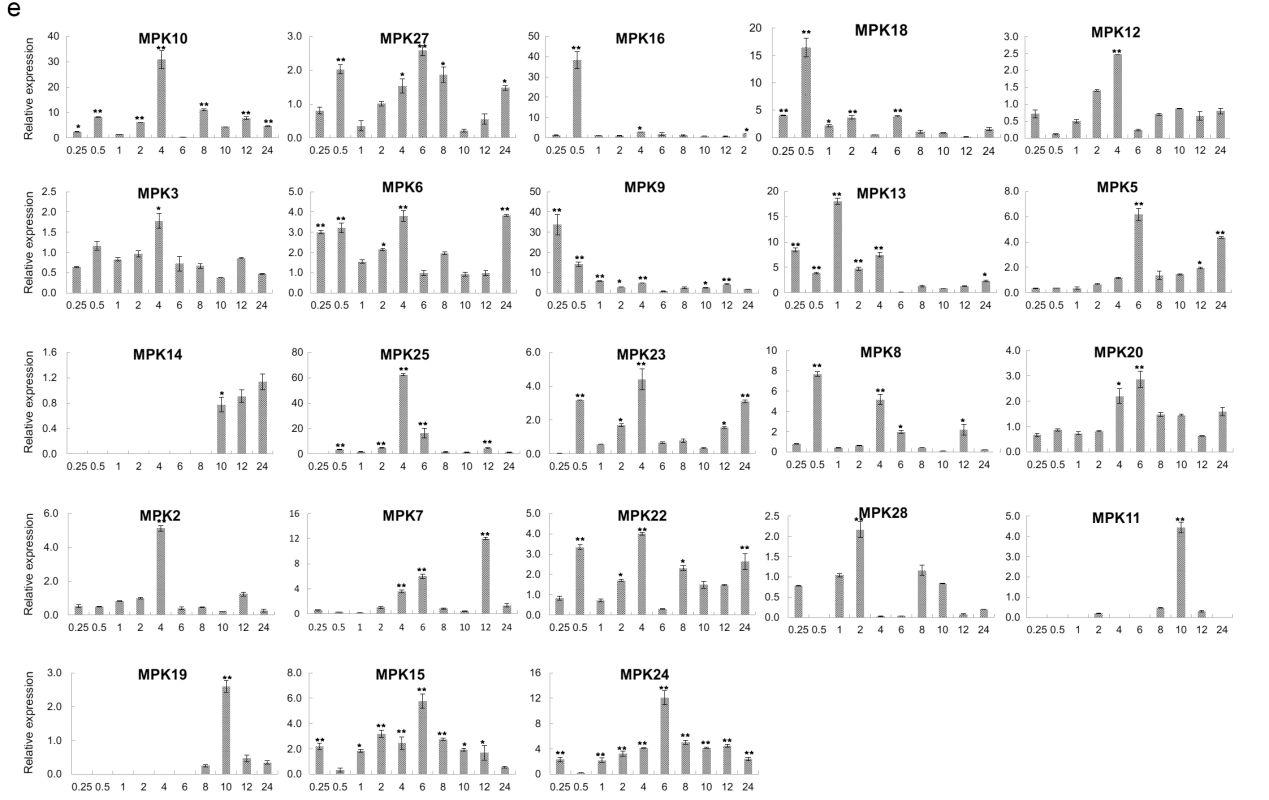

Supplement: Additional file 7: Figure S3. — Expression patterns of MAPK genes under stress treatments (a, NaCl; b, PEG; c, 4°C; d, 37°C; e, wounding). The expression levels data were presented as the mean fold by comparing experiments and controls samples. The Y-axis indicates relative expression levels and the X-axis indicates the hours of stress treatments. The error bars were calculated based on three biological replicates using standard deviation. “*”: significant difference (P < 0.05); “**”: significant difference (p < 0.01). [file 12870_2014_345_MOESM7_ESM.docx]
